# Nanostructured Free‐Form Objects via a Synergy of 3D Printing and Thermal Nanoimprinting

**DOI:** 10.1002/gch2.201800083

**Published:** 2018-12-03

**Authors:** Jumiati Wu, Wei Li Lee, Hong Yee Low

**Affiliations:** ^1^ Digital Manufacturing and Design Centre (DManD) Engineering Product Development (EPD) Singapore University of Technology and Design 8 Somapah Road 487372 Singapore

**Keywords:** 3D printing, curved object, nanoimprint lithography, nanoinjection molding, nanostructured 3D mold insert

## Abstract

High‐resolution surface patterning has garnered interests as a nonchemical‐based surface engineering approach for creating functional surfaces. Applications in consumer products, parts for transportation vehicles, optics, and biomedical technologies demand topographic patterning on 3D net shape objects. Through a hybrid approach, high‐resolution surface texture is incorporated onto 3D‐printed polymers via direct thermal nanoimprinting process. The synergy of geometry design freedom in 3D printing and the high spatial resolution in nanoimprinting is demonstrated to be a versatile fabrication of high‐fidelity surface pattern (from 2 µm to 200 nm resolution) on convex, concave semicylindrical, and hemispherical objects spanning a range of surface curvatures. The novel hybrid fabrication is further extended to achieve a high‐resolution curved mold insert for rapid prototyping via injection molding. The versatility of the fabrication strategies reported here not only provides a post‐3D printing process that enhances the surface properties of 3D‐printed objects but also opens a new pathway to enable future study on the effects of combining microscale and nanoscale surface texture with macroscopic curvature. Both have been known, individually, as an effective approach to tune surface functionalities.

## 1 Introduction

High‐resolution surface patterning has evolved from being a mainstream lithography for semiconductor device fabrication to be a new surface engineering technique for creating functional surfaces.^1^, ^2^, ^3^ Compared to traditional surface roughening approaches, highly deterministic surface patterning techniques have provided an unprecedented design freedom to physical surface engineering. Highly precise, ordered, and uniform surface patterns that cut across the micrometer and nanometer length scales offer a high degree of tunability for physical surface engineering. Surface properties such as wettability,^4^, ^5^ friction/adhesion,^2^ and optical effects^6^ are some of the most frequently encountered properties in applications ranging from biomedical products^7^ (sensors, diagnostic devices) to optoelectronic devices^8^, ^9^ and consumer products.^10^, ^11^, ^12^ Highly deterministic surface textures that lead to highly tunable surface properties offer a promising surface engineering approach for multiple reasons: 1) preserves the native chemical structure of the materials, 2) minimizes the use of chemical, thus offering a more sustainable approach for surface engineering, 3) multiple surface properties can be achieved through surface texturing, and 4) in nanometer scale surface texture, optical transparency of the material can be preserved. The multifaceted benefits are highly desirable in new product developments.

High‐resolution functional surface patterns have been achieved primarily via lithography processes. Lithography is inherently a 2D process, adaptable only to flat and planar formats such as wafer, foil, films, and sheets. As a result, the existing body of literature on surface texture enabled functionalities is limited to flat and planer formats. Unlike 2D substrates, 3D objects are complicated with a high degree of variations in nonplanarity and/or curvatures. Among the established lithographic processes, nanoimprint lithography has been recently shown to achieve high resolution surface patterning on quasi‐3D substrates. A few publications have reported nanoimprinting of curved substrate using soft and conformal mold, such as poly(dimethylsiloxane) (PDMS)^13^, ^14^, ^15^, ^16^, ^17^ and polyurethane acrylate (PUA).^18^ For example, PDMS mold has been used in nanoimprint lithography (NIL) process to pattern micron or submicron features down to 300 nm on polymer‐coated curved substrates at radius of curvature (*R*) between 20 and 92.5 mm.^13^, ^14^, ^15^, ^16^, ^17^ A drawback of PDMS elastomeric mold is the relatively low Young's modulus of 3 MPa,^19^ which imposes a limit on pattern resolution. Recently, PUA bilayer mold comprising a soft backing and a hard pattern‐carrying layer was reported for patterning curved surfaces with resolution down to 80 nm.^18^ Another recent work demonstrated nanostructuring of 3D structure by combining NIL and 3D projection stereolithography.^20^ 3D scaffolds were fabricated layer by layer via projection stereolithography while nanostructures are simultaneously fabricated by placing nanoimprinted PDMS mold on vat bottom. A challenge that the above technique faces is the undesirable polymerization of liquid resin in between scaffold layers due to stray light and accumulation of exposure dose overtime. In general, these prior works demonstrated some level of success but in most instances with limited design flexibility in material selection and complexity of geometry.

Additive manufacturing process such as 3D printing enables the direct fabrication of net shape objects. Despite the rapid advancement in 3D printing of polymers, commercial 3D printers lack the resolution capability of lithographic processes.^21^ On the other hand, lithographic processes are limited to planar 2D objects, except for two‐photon polymerization process.^22^ The later, however, is a relatively expensive research tool, with limited part size scalability. In this work, we combined the advantage of geometry freedom in 3D printing with high resolution capability in nanoimprinting to fabricate high resolution surface topography directly on a net shape object, the product of which was subsequently investigated as a mold insert for replication via nanoinjection molding.

Injection molding is a universal manufacturing process for the production of 3D objects with a high degree of freedom in geometry complexity. 3D objects ranging from simple consumer products to sophisticated electronic, automotive, and biomedical products are made by injection molding in the millions. Since injection molding is a replication process, the resolution and the geometry of molded products are, in priori, defined by the mold inserts. High resolution dimension is well established in the injection molding of compact disc (CD) and digital video disc (DVD), which are flat and planar products.^23^, ^24^, ^25^ Similarly, the existing publications on injection molding of polymer micro‐ and submicron structures were achieved using mold inserts fabricated by 2D lithographic processes,^25^, ^26^, ^27^ meaning that the geometry of the produced parts are also limited to flat and planar format. Injection molding of sub‐100 nm nanostructures with aspect ratio of 2.5 pillars using flat nickel mold inserts has been reported.^25^ A rapid prototyping technique for the fabrication of injection mold insert is 3D printing process.^28^, ^29^ 3D printing process produces highly customizable 3D object designed by 3D digital model. High specification 3D printing tool has achieved a resolution of about 20 µm,^30^, ^31^ while majority of the commercially available 3D printing processes/tools are still plugged by the inherently rough surface finishing.^32^, ^33^ Two‐photon‐lithography, specifically femtosecond laser–induced photopolymerization of resin, is a promising 3D fabrication technique with resolution reported to achieve 200 nm.^34^ The throughput of two‐photon photopolymerization fabrication of 3D object is low due to the point form writing scheme of the process, consequently the process is limited to small size object, typically no more than 50 mm.^35^, ^36^ Hence, challenges are still present to achieve scalable high resolution surface patterning on 3D objects. Here, we report a rapid prototyping technique that combines 3D printing and nanoimprinting to achieve high resolution surface patterning on nonplanar mold insert for injection molding. Additionally, injection molding has been conducted to produce curved objects with sub‐micron surface textures.

## 2 Result and Discussion

### 2.1 Nanoimprinting on 3D‐Printed Nonplanar Objects

Using the PolyJet 3D printer, a series of semicylindrical and hemispherical objects with a systematic variation in the curvature were fabricated. PolyJet prints 3D objects using photocurable resins. A pre‐requisite in choosing the 3D printing process is that the 3D‐printed materials have a glass transition temperature (*T*_g_) or thermal deformation temperature within the typical processing window of nanoimprinting. Two types of materials were used in this study: VeroClear (*T*_g_: 52–54 °C) and Digital‐ABS (*T*_g_: 47–53 °C). In particular, Digital ABS has been widely used for 3D printing of mold inserts due to its long tool life (80 or more shots with elastomeric plastics) and suitability for molding complex geometries or molding with higher temperature thermoplastics.^37^, ^38^ In nanoimprinting process, the surface to be imprinted is referred to as the substrate. Here, the 3D‐printed curved objects are referred to as curved substrates.

**Figure 1:**
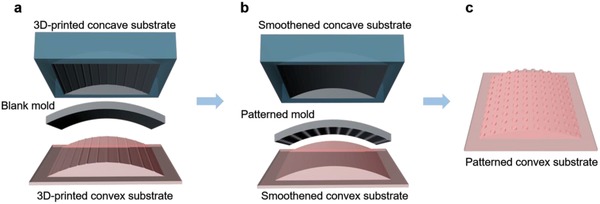
Schematic diagram (not to scale) of a two‐step thermal nanoimprinting process on a curved 3D‐printed mold insert. a) Step 1: Eliminate surface waviness of 3D‐printed object by smoothening process with smooth PC film. b) Step 2: Pattern transfer by thermal nanoimprinting process with patterned soft polymeric mold. c) Nanoimprinted surface pattern on the curved object.

A two‐step thermal NIL was introduced to fabricate micro‐ and sub‐micrometer features on curved 3D‐printed objects as shown in **Figure** **1**.

*Step 1. Surface smoothening*. An inherent drawback of today's 3D printing process is the considerably high surface waviness, typically in the tens or even hundreds of micrometer. Surface waviness along the curvature of curved substrate was imaged with digital microscope. Its optical cross‐sectional view and measured 3D surface profile (on curved substrates of *R* = 9 mm) can be found in Supporting Information. Surface waviness acts as an obstacle for conformal contact between a high resolution mold and the imprint material. Failure to remove surface waviness will result in incomplete pattern transfer during nanoimprinting. A surface‐smoothening process was thus necessary before nanoimprinting can be performed on the curved substrates. Using optical grade polycarbonate (PC) sheet, surface smoothening was achieved via a nanoimprinting process. The optical grade PC sheet replaced the ‘mold' in a typical nanoimprint material stack. Nonpatterned or blank PC sheet was sandwiched between a concave and a convex 3D‐printed parts during imprint (Figure 1a). Representative cross‐sectional view and measured 3D surface profile of smoothened curved substrates with *R* = 9 mm can be found in Supporting Information. Surface waviness was significantly reduced after the smoothening step.

**Figure 2:**
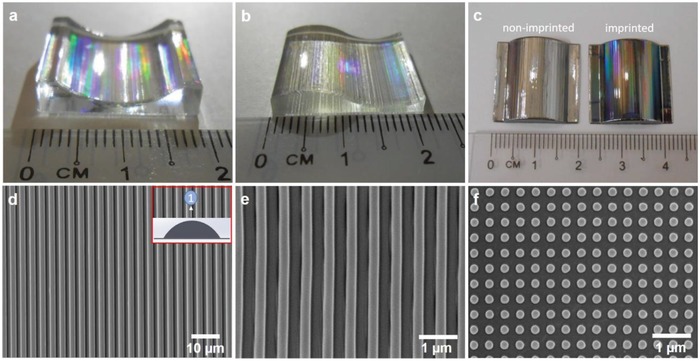
Photograph of imprinted a) concave, b) convex–concave 3D‐printed substrate with *R* = +/−10.9 mm, c) imprinted and nonimprinted convex substrate (*R* = 9 mm) with Au‐Pd coating. d–f) SEM images of imprinted convex 3D‐printed part with various *R* captured at position 1, as shown in inset d. Representative SEM images of nanoimprinted VeroClear with d) 2 µm line gratings on curved object with *R* = 28.6 mm, e) 300 nm line gratings on curved object with *R* = 9 mm, f) 200 nm cylindrical pillars on curved object with *R* = 10.9 mm. *R* refers to radius of curvature.

*Step 2. Nanoimprinting on curved substrates*. One requirement of a successful surface pattern replication by nanoimprinting is that the mold is in conformal contact with the imprint material. In standard nanoimprinting material stack, the mold and the imprint film (either coated on a flat rigid substrate or a freestanding polymer film) are both flat and planar; mold conformation to the imprint film is achieved uniformly through a uniform pressure application. The 3D‐printed semicylindrical object investigated here presents a level of complexity for nanoimprinting process. Along the length of the semicylindrical substrate, the curvature is zero; hence this substrate has only one curvature. However, at high degree of surface curvature, uniform mechanical conformity between nanoimprint mold and the semicylindrical substrate is difficult to achieve; hence, existing literature on nanoimprinting of curved substrates reports limited curvature variations. The challenges in nonplanar substrate nanoimprinting are further amplified for hemispherical substrate, which presents an additional level of difficulty due to the isotropic curvature over the entire surface. In this work, a pair of geometrically matching curved substrates was used to mitigate conformity limitation. The current approach contrasts an earlier approach called dual‐mold nanoimprinting where a top and bottom silicon molds are used to nanoimprint complex 3D nanostructures on flat substrate.^39^, ^40^ Here, ethylene tetrafluoroethylene (ETFE) was used as the soft and flexible mold material due to its relatively high modulus (*E* = 1 GPa), its stability at high temperature (melting point, *T*_m_, 240–280 °C) as well as its anti‐sticking properties (surface energy, 15.6 dyn cm^−1^).^41^, ^42^ ETFE mold was fabricated from master Si mold via thermal NIL, which is described in Experimental Section. Similar to the nanoimprinting step described in step 1, patterned ETFE was sandwiched between the smoothened concave and convex 3D‐printed parts (Figure 1b). Successful imprint on the curved substrate was visually apparent by the rainbow colors caused by optical diffraction (**Figure** **2**a,b). Imprinted and nonimprinted curved substrates are also visually distinguishable with a sputtered layer of Au‐Pd (Figure 2c). Representative SEM images taken at the peak of the semicylindrical objects are shown in Figure 2d–f. Two micrometers gratings on *R* = 28.6 mm (Figure 2d), 300 nm gratings on *R* = 9 mm (Figure 2e), and 200 nm pillars *R* = 10.9 mm (Figure 2f) show high imprint fidelities. The overall patterning yield is defined as the area percentage of pattern transfer from the mold to the curved substrate. Here, the yield was estimated by the ratio of the cross‐sectional arc length between the imprinted curved object and the flexible mold, as below:

(1) $$\mathrm{Yield}=\frac{L_{3D}}{L_{\mathrm{mold}}}\times 100\%$$

where *L*_3D_ is arc length of imprinted 3D‐printed part and *L*_mold_ is arc length of flexible mold. A schematic of yield calculation can be found in Supporting Information. Using the above method, the overall pattern transfer or imprinting yield was estimated to be >93%.

**Figure 3:**
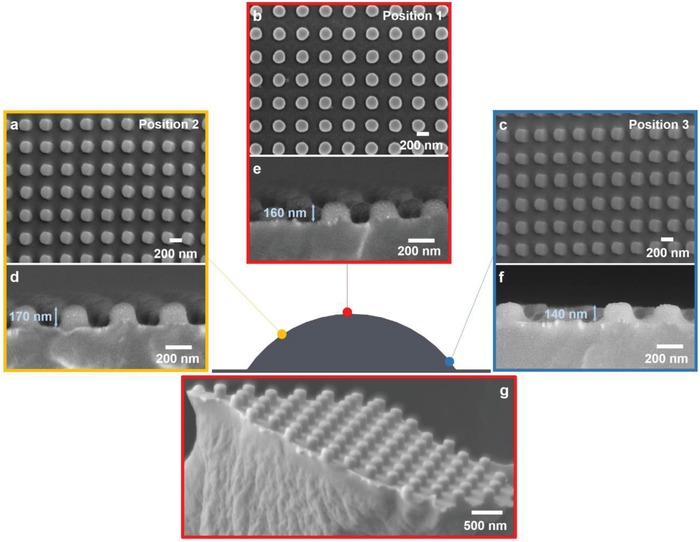
SEM images of 200 nm cylindrical pillars imprinted on curved 3D‐printed object with radius of curvature, *R* = 10.9 mm. a–c) Top view and d–f) cross‐sectional view of imprinted 3D object at three different positions along the curvature. g) Tilted view of imprinted 3D object at position 1 marked by red dot.

A high yield of pattern transfer is a priori governed by the macroscopic conformation of the soft mold over the curved substrates; however it is not an indication of the fidelity of the micro‐ and sub‐micrometer structure across the imprint field. A high imprint fidelity requires a uniform temperature and pressure application across the imprint field during the nanoimprinting process. Due to the curvature of the substrate, pressure uniformity across the curvature is compromised, generally with the side of the curvature experiencing a lower pressure than the top of the curvature. Two hundred nanometers diameter pillar structure (aspect ratio of 1:1) imprinted on the *R* = 10.9 mm object was used to investigate the fidelity of the imprints along its curvature. Top and cross‐sectional SEM views at three different positions along the curvature (represented by colored dots along curvature) are shown in **Figure** **3**. Top view SEM images are shown in Figure 3a–c, while cross‐sectional scanning electron microscope (SEM) views are shown in Figure 3d–f. Top view shows that uniform lateral dimension is replicated along the curvature. Average lateral dimension of 203 ± 4 nm, 206 ± 6 nm, and 205 ± 4 nm was measured for three locations along the curvature. Lateral dimension variation of <5% indicates a high fidelity replication of cylindrical pillars on convex 3D‐printed objects. However, variation in pillar height is observed in the cross‐sectional SEM images. Average pillar height of 158 ± 9 nm, 170 ± 10 nm, and 143 ± 7 nm was measured on the same three locations along the curvature. The maximum pillar height reached 85% of the full height of surface relief structure on the mold. The discrepancy in the pillar height is due to incomplete filling during the nanoimprinting process caused primarily by a lower pressure application at the side of the curvature. A tilted view of the *R* = 10.9 mm convex object was obtained from a fractured sample (Figure 3g), showing an array of uniform nanopillars. The patterning process via the two‐step thermal NIL has successfully achieved high density features with resolution of 200 nm on curved substrate across greater than 110° arc of curvature. Representative SEM images of other imprinted objects with various radius of curvature can be found in Supporting Information.

**Figure 4:**
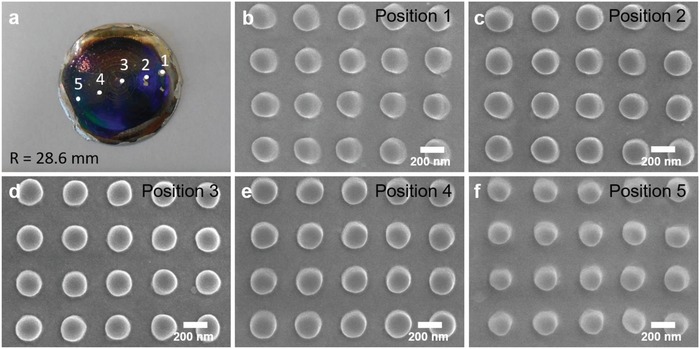
a) Photograph and b–f) SEM images of top view of 200 nm cylindrical pillars imprinted on a hemispherical 3D‐printed object (VeroClear) with radius of curvature, *R* = 28.6 mm at five different positions.

**Figure 5:**
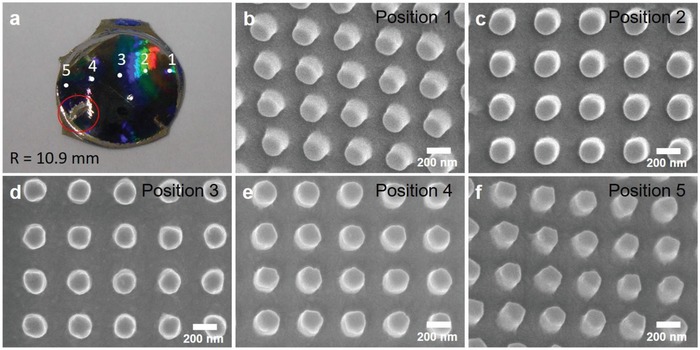
a) Photograph and b–f) SEM images of top view of 200 nm cylindrical pillars imprinted on a hemispherical 3D‐printed object (VeroClear) with radius of curvature, *R* = 10.9 mm at five different positions.

When a soft mold conforms to the curvature of a nonplanar substrate, the soft mold undergoes a strain deformation, potentially resulting in surface pattern deformation. On the semicylindrical concave substrates, the surface pattern on the soft mold would undergo unidirectional compressive strain deformation along the direction of the curvature. The extent of strain deformation is directly dependent on the degree of curvature. Due to a considerably large radius of curvature (a range of few tens of millimeters) compared to the sub‐micron scale surface structure, no appreciable strain deformation was transferred from the soft mold to the semicylindrical substrates. However, on hemispherical substrate, there is a limitation to the conformability between the soft mold and the curved substrate beyond which folding of the soft mold compromises the surface imprinting yield. On hemispherical substrate with lightly curved substrate (*R* = 28.6 mm), the geometrical area difference between a 2D soft mold and a 3D curved substrate is minimum such that the soft mold is able to conform to the curved substrate. Homogenous diffractive reflection which represents the existence of submicron and micron‐sized feature is observed throughout the surface after Au‐Pd coating (**Figure** **4**a). Feature of 200 nm diameter pillar is replicated onto the hemispherical substrate with representative images taken from five different positions along the curvature as observed in Figure 4b–f. The soft mold's ability to conform to curved substrate results in no substantial deformation of pattern features. However, on highly curved substrate, to counter offset the geometrical area difference of 2D mold and 3D curved substrate, nanoimprinting on hemispherical substrate was achieved by making a cut on the soft mold. Red circle on **Figure** **5**a indicates nonimprinted region of 3D hemispherical object, due to uneven cut edges on the soft mold. Figure 5b–f shows representative top view SEM images of 200 nm pillar nanoimprinted on hemispherical 3D printed VeroClear at *R* = 10.9 mm. Note that top view SEM images at position 1, 2, 4, 5 appear to be tilted at an angle due to curvature of the substrate. Circular pillar feature is maintained at position 1–4, however at position 5; polygonal‐like feature is observed instead of circular feature. This is due to lack of conformality of ETFE soft mold on hemispherical curved substrate at position 5, near the cut edges. In order to achieve complete pattern replication on more complex 3D substrate further study is underway. The nanoimprinted 3D‐printed nonplanar substrate can subsequently be used as mold insert for nanoinjection molding.

### 2.2 Nanoinjection Molding of High Resolution Surface Texture on Curved Objects

**Figure 6:**
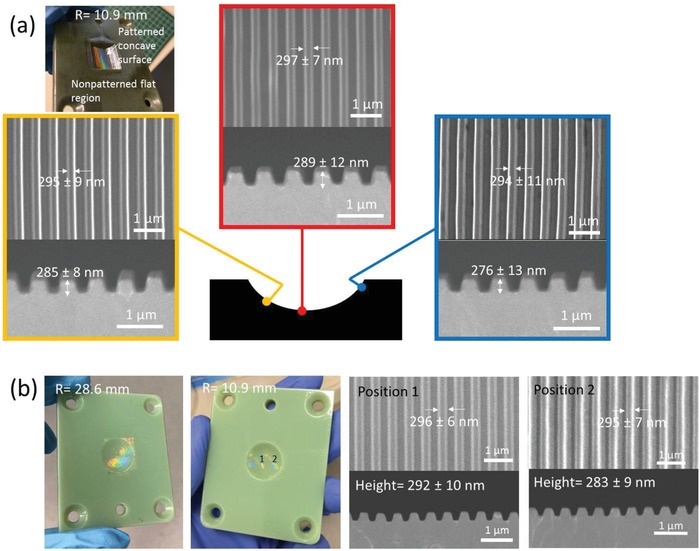
a) SEM images of top and cross‐sectional views of 300 nm grating imprinted on a curved (semicylindrical concave) 3D‐printed mold insert with radius of curvature (*R*) of 10.9 mm at three different positions along the curvature. Photograph of a nanoimprinted mold insert coated with Au‐Pd is shown in the top left. b) Photographs of mold inserts with hemispherical concave surfaces with *R* = 28.6 and 10.9 mm, and the corresponding SEM images of top and cross‐sectional views of 300 nm grating imprinted on the mold insert (*R* = 10.9 mm) at two different positions along the curvature.

Digital ABS is a relatively stable polymer often used as a mold insert for prototyping by injection molding process. **Figure** **6** shows representative results of nanoimprinted curved mold inserts in Digital ABS. Similar to VeroClear, the nanoimprinting yield and pattern fidelity of the 300 nm grating structure were >90%. High flow PP (COSMOPLENE AX 161) with *T*_g_ of −7 °C, melt flow rate (ASTM D1238) of 65 g/10 min and shrinkage of 1% was used for injection molding. The PP has a melting point at 160 °C, and the melt temperature was set at 210 °C to prevent the formation of knit lines.

**Figure 7:**
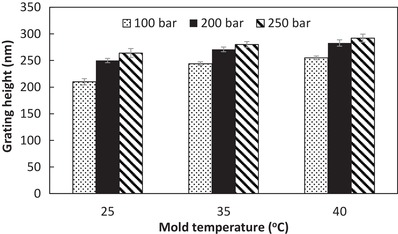
Grating height on the center of semicylindrical convex surface (*R* = 10.9 mm) as a function of mold temperature for various holding pressures.

**Figure 8:**
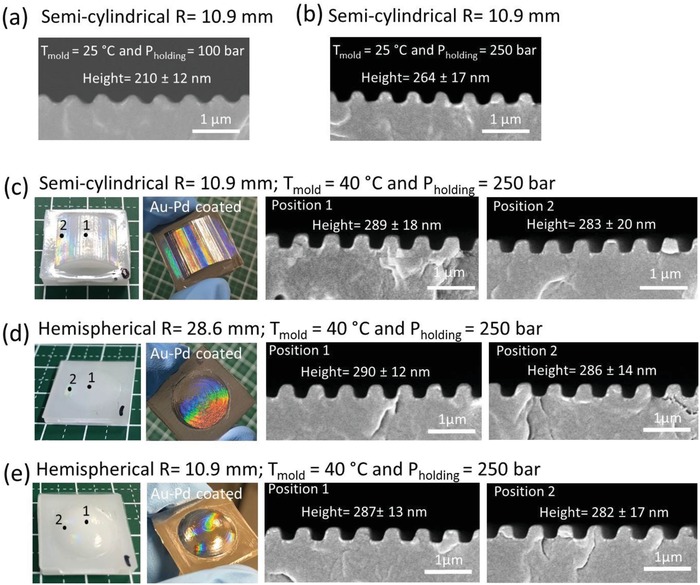
a–b) SEM images of cross‐sectional views of injection‐molded grating structure on semicylindrical convex part fabricated by different process parameters. c–e) Photographs of injection‐molded parts (uncoated and coated with Au‐Pd) with two types of curved surfaces: semicylindrical and hemispherical convex, and their respective SEM images of cross‐sectional views of injection‐molded 300 nm grating at two different positions along the curvature.

The nanoimprinted curved substrate serves as a prototype mold insert for injection molding. In this work, the grating orientation was designed to be aligned with the polymer melt flow to promote filling of the cavity. It had been reported that the air in the grating cavity aligned with the flow direction could partially escape from the cavity, whereas the escape of air is limited when the grating is aligned orthogonal to the flow direction.^27^, ^43^ To investigate the influence of process parameters on the replication fidelity, the range values for mold temperature (*T*_mold_) and holding pressure (*P*_holding_) were defined considering the thermal and mechanical properties of the mold insert's material and injection molding plastic material. In the current work, the relatively low range values for mold temperature (25–40 °C) and holding pressure (100–250 bar) were used for injection molding of sub‐micron surface patterns in comparison with other works (*T*_mold_ > 100 °C and *P*_holding_ > 700 bar),^24^, ^26^, ^43^ in which the mold inserts were made of nickel shim or silicon. For polymeric mold inserts, patterned structure on the mold underwent deformation when the mold temperature was above 50 °C (i.e., the *T*_g_ of Digital ABS mold material), whereas a pronounced flash along parting lines was formed when a holding pressure above 400 bar was applied (as shown in Figure S4, Supporting Information). A balance between achieving complete filling of polymer in mold cavity and the thermomechanical stability of the mold inserts has been achieved in this work. The process optimization results indicated that the grating height increases when the mold temperature and holding pressure are increased (*p* < 0.05) (**Figure** **7**). For instance, as shown in **Figure** **8**a, the grating on a semicylindrical (*R* = 10.9 mm) convex part was poorly replicated with low grating height (210 ± 12 nm) and rounded top when the room temperature of 25 °C was applied to the mold and a holding pressure of 100 bar was used. The replication was improved, but the grating height of 264 ± 17 nm was still 10% shorter than the mold dimension when the holding pressure was increased to 250 bar but mold temperature remained at 25 °C (Figure 8b). The highest replication quality in terms of grating height, width, and shape is achievable for *T*_mold_ = 40 °C and *P*_holding_ = 250 bar (Figure 8c). The tops of the 300 nm grating on the semicylindrical convex part (*R* = 10.9 mm) were in the form of squarish shape, which is an indication of complete filling into surface cavities of the mold. A good pattern transfer (>95% of the mold dimension) with average grating height of 289 nm and width of 295 nm is achieved at positions 1 (center) and 2 (side). Iridescent color was also observed to cover the entire convex surface, suggesting nearly 100% yield of pattern transfer. In comparison, the nonpatterned convex surface of an injection‐molded object shows no iridescent color. Similarly, the same process parameters were able to produce 300 nm grating with high pattern yield and fidelity for the hemispherical parts (*R* = 10.9 and 28.6 mm) (Figure 8d,e).

In injection molding process, filling of sub‐micron or nanostructures is thermally limited by the extremely fast solidification (nanoseconds to microseconds) of polymer melt coming into contact with a relatively cold mold wall.^44^, ^45^ As a result, during filling stage, the polymer melt fills the macroscopic cavity while stagnating at the entrance of sub‐micron cavities. Full replication of features, in particular sub‐micron or nanoscale cavities, requires pressure‐driven deformation of a frozen layer during packing stage.^27^, ^46^ During the packing stage, the thickness of the frozen layer can be reduced by reducing the thermal gradient between the frozen layer and the mold wall. At a higher mold temperature, a thinner frozen layer is easier to be deformed into the sub‐micron cavities under an elevated packing pressure. To fill the micro/ nanocavities successfully, the mold temperature is normally set greater than the *T*_g_ of the polymer used. PP with *T*_g_ of −7 °C and high melt flow rate used in this work is to ensure high polymer chain mobility and flowability to fill the sub‐micron cavities. The polymer fills the surface cavity corner if the pressure exerted on it overcomes the entrapped air pressure and capillary pressure. The effect of the capillary pressure is significant only if the dimension is smaller than 100 nm,^47^ hence it can be neglected from sub‐micron feature replication. When the polymer fills the cavities, the entrapped air is compressed and the input pressure counteracts, delaying the progression of filling. A high holding pressure overcomes the entrapped air pressure, promoting the polymer filling in the surface cavities.

Complete filling of micro/nanocavity is not the only factor for achieving high replication fidelity as the molded structures must also withstand demolding. Demolding can exert high frictional forces on surface features, which may lead to structural failure of the replicated surface structures. Anti‐stick coatings on patterned mold used in hot embossing and nanoimprint lithography have been shown to reduce polymer stiction during demolding.^48^, ^49^ In this work, 1H,1H,2H,2H‐perfluorodecyltrichlorosilane (FDTS) coating on the mold inserts was used to preserve the initial mold cavity filling structure during demolding process. The grating pattern of aspect ratio of 1:1 on the convex surface used in this work can be preserved after demolding. However, the aspect ratio and degree of curvature may be the important parameters that affect the quality of replicated features during demolding; to fully understand this phenomenon would be a subject of a future investigation.

## 3 Conclusions

The challenge of fabricating high‐resolution surface topography on free‐form objects has been overcome via a hybrid prototyping technique which combines the free‐form fabrication capability of 3D printing and the high surface resolution capability of nanoimprinting. 3D‐printed semicylindrical and hemispherical objects with radius of curvature ranging from ± 9 to ± 28.6 mm were directly nanoimprinted with surface pattern of 2 µm to 200 nm. High pattern fidelity (85%) and high yield (93%) of imprinted patterns (with resolution down to 200 nm) along the surface curvature have been achieved. The technique developed in this work has overcome the inherent drawbacks of the rough surfaces in 3D printing and the limitation of nanoimprinting for free‐form objects, hence it is also a post 3D printing process to enhance or augment the surface properties of 3D‐printed polymers.

The nanoimprinted 3D‐printed polymer was demonstrated as a rapid prototyping mold inserts for nanoinjection molding. Injection‐molded semicylindrical and hemispherical PP with surface topography consisting of 300 nm grating was achieved with nearly 100% yield and pattern fidelity >95% (2.5 cm^2^ in surface area). This work paves the way for future studies and discoveries of novel surface properties through the combined effect of macroscopic curvature and high‐resolution surface texture. The process developed here can serve as a rapid prototyping technique for product design and pilot scale process development.

## 4 Experimental Section

*3D Printing of Curved Objects*: 3D curved objects were designed with 3D CAD design software (SOLIDWORKS) with dimension of 2 × 2 cm (*L* × *W*) with varying radius of curvature (no curvature, +/− 9, 10.9, 15.1, and 28.6 mm). Similarly, the two‐plate mold inserts were designed with dimension of 6 × 5 cm in size and a thickness of 4.5 cm for the cavity plate and 10 cm for the core plate. The design consideration of mold inserts is described in the Supporting Information. The curved surface (1.5 × 1.5 cm) with radius of curvature (*R* = 10.9 or 28.6 mm) was at the center of a mold insert. The 3D CAD file was then fed into a 3D printer (Stratasys Objet30 Prime) for printing with rigid and transparent material (RGD810 VeroClear) or into a multimaterial 3D printer (Stratasys J750) for printing with ABS‐like material (“Digital ABS” a mixture of RGD515 and RGD535). The 3D objects and mold inserts were printed glossy finish on top surface. Print lines were along the direction of injection flow path. The support material (SUP705) was removed manually, and the printed parts were rinsed with water thoroughly and kept in dry box prior to usage.

*Thermal Nanoimprinting of Patterned Flexible Mold*: Silicon (Si) master molds with various features were purchased from EULITHA. Si molds were cleaned with piranha solution which contained a mixture of 96% sulfuric acid and 30% hydrogen peroxide in 3:1 ratio at 120 °C for 1 h. Si master molds were then coated with anti‐stick layer of FDTS using Nanonex NX‐VT15 at 80 °C for 1 h prior to feature replication onto flexible polymer film. A flexible polymer film, Fluon ETFE film with thickness of 100 ± 10 µm was purchased from Asahi Glass Co. Patterning of polymeric sheets was performed via thermal nanoimprint lithography (NIL). The polymeric sheet was placed on treated Si mold containing intended features. ETFE film was imprinted using Nanonex NX‐2006 with temperature of 240 °C and pressure of 40 bar for 5 min. Imprinted ETFE with the desired structures were subsequently used as flexible mold in 3D imprint lithography without further processing.

*Nanoimprinting of Sub‐Micron Surface Texture on 3D Curved Objects and Mold Inserts*: Polymeric 3D‐printed curved objects and mold inserts with sub‐micron surface texture were fabricated with a two‐step thermal nanoimprint lithography. The first step, a surface‐smoothening process. An optical grade, smooth PC film (thickness of 125 µm, purchased from INNOX), was sandwiched between 3D‐printed substrates and imprinted at 100 °C with pressure of 20–30 bar for 5 min using a nanoimprinter (Nanonex NX‐2006). The system was then cooled down to the temperature below 35 °C prior to demolding. In the second step, surface patterning on 3D‐printed object, patterned ETFE sheet was used as a flexible mold. The imprint was subsequently performed at 120 °C with pressure of 30–50 bar for 5 min. The nanoimprint system was then cooled down to the temperature below 35 °C before demolding.

*Injection Molding of Curved Objects with High Resolution Surface Pattern*: Polymer replicas were injection molded through a microinjection molding machine (BABYPLAST 6/10P) using commercially available polypropylene (PP, COSMOPLENE AX 161). PP was baked at 80 °C for 6 h to remove the moisture just before it was used in injection molding. The steel mold base was designed to hold the mold insert in place at the center, where the insert could be easily replaced as needed. All moldings were performed with a constant flow rate at 6.157 cm^3^ s^−1^ (that gave rise to a filling time of about 0.4 s) under injection pressure of 200 bar, a holding time of 10 s, and barrel temperature of 210 °C. The influences of mold temperature and holding pressure on the replication fidelity were explored separately, while keeping all other parameters constant. Due to the low thermal conductivity of the polymer‐based mold insert, ample time between shots was needed to allow the mold to achieve thermal equilibrium at targeted temperature monitored using a solid probe thermometer. Fifteen parts were injection molded, with only the last three parts in each series being used for the characterization to assure stabilization of the molding process.

*SEM Imaging of Surface Patterns*: The analysis of the samples was conducted through a field emission SEM (JEOL FESEM, JSM 6700F). Prior to SEM imaging, all samples were deposited with a thin layer of Au:Pd/80:20 (≈8 nm) (Fine Coater, JEOL JFC‐1600) under plasma current of 30 mA for 35 s. For every sample batch (*n* = 3), since morphologies were found to be consistent, only one representative SEM image is shown. Dimensions of surface pattern (mean ± standard deviation for *n* > 5 measurements) in independent SEM images were analyzed with ImageJ software. The curved 3D‐printed object's surface profile was imaged by digital optical microscope (Hirox KH‐8700).

## Conflict of Interest

The authors declare no conflict of interest.

## Supporting information

Supplementary
