# Supplementary material for: Nanostructured Free‐Form Objects via a Synergy of 3D Printing and Thermal Nanoimprinting
Source: Glob Chall. 2018 Dec 3;3(5):1800083. doi: 10.1002/gch2.201800083 (PMC6498116; doi:10.1002/gch2.201800083)
Supplement: Supplementary file 1 — Supplementary [file GCH2-3-1800083-s001.pdf]

Copyright WILEY-VCH Verlag GmbH & Co. KGaA, 69469 Weinheim, Germany, 2018.

Supporting Information

for *Global Challenges,* DOI: 10.1002/gch2.201800083

Nanostructured Free-Form Objects via a Synergy of 3D Printing and Thermal Nanoimprinting

*Jumiati Wu, Wei Li Lee, and Hong Yee Low**

Supporting Information

Nanostructured Free-form Objects via a Synergy of 3D Printing and Nanoimprinting

*Jumiati Wu,† Wei Li Lee† and Hong Yee Low**

Engineering Product Development, Singapore University of Technology and Design, 8 Somapah Road, 487372 Singapore.

Digital Manufacturing and Design Centre, Singapore University of Technology and Design, 8 Somapah Road, 487372 Singapore.

† These authors contributed equally to this work.

Design consideration of the mold insert

The thicknesses of inserts were designed to extend beyond the face of a Master Unit Die (MUD) base by 0.2 mm to establish parting surfaces that will have minimal flash. Sprue bushings were included in the MUD base to prevent direct nozzle contact with the mold insert. Fan gate was introduced to improve the material flow with a minimum of pressure loss. A draft angle of 5° was added to the vertical sides to facilitate molded part removal.

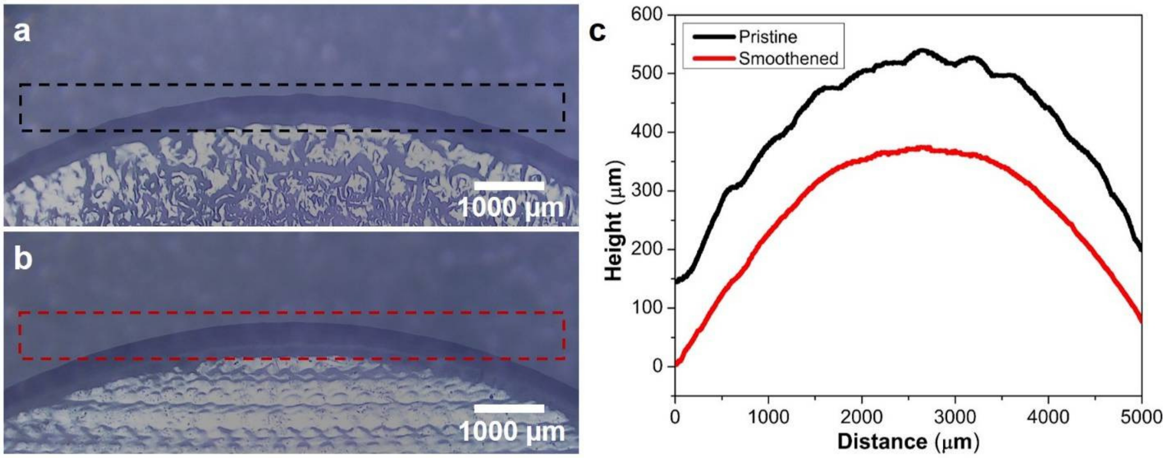

Figure S1. 3D printed curved objects' surface profiles. Cross-sectional view of (a) as-printed/pristine and (b) smoothened 3D printed curved objects (R= 9 mm) imaged with

optical microscope. (c) Surface profiles of both pristine (black line) and smoothened (red line) 3D printed curved objects.

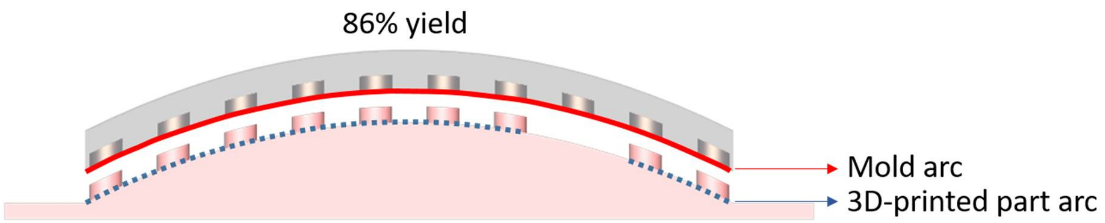

Figure S2. Cross-sectional schematic images of thermal NIL process on curved object with partial yield.

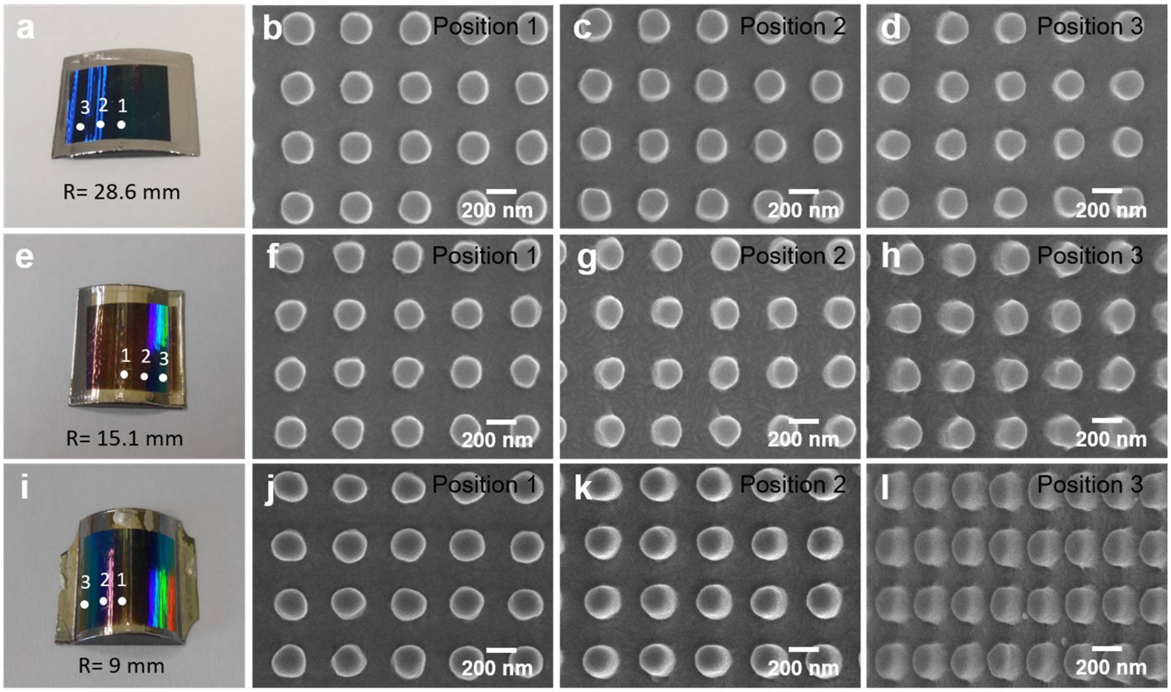

Figure S3. Representative photograph and SEM images of top view of 200 nm cylindrical pillars imprinted on a 3D-printed object (Veroclear) with radius of curvature, R= 28.6 mm (a-d); R= 15.1 mm (e-h); R= 9 mm (i-l) at 3 different positions.

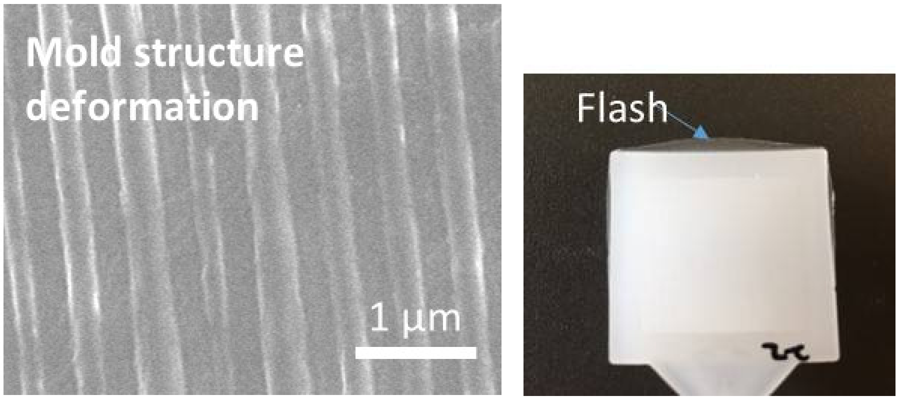

Figure S4. SEM image of deformed mold structure (left) and photograph of flash formation along parting lines (right).
